# Blood Pressure Management Following Large Vessel Occlusion Strokes: A Narrative Review

**DOI:** 10.4274/balkanmedj.galenos.2020.2020.4.196

**Published:** 2020-08-11

**Authors:** Saurav Das, Kevin Denny John, Satheesh Kumar Bokka, Kerri Remmel, Ozan Akça

**Affiliations:** 1 Department of Neurology, Louisville University School of Medicine, Louisville, Kentucky, USA; 2 University of Louisville, School of Medicine, Louisville, Kentucky, USA; 3 Department of Anesthesiology and Perioperative Medicine, Stroke ICU, Louisville University Hospital, Louisville, Kentucky, USA; 4 Comprehensive Stroke Clinical Research Program, University of Louisville, Louisville, Kentucky, USA

**Keywords:** Blood pressure variability, cerebral autoregulation, endovascular thrombectomy, hypertension, ischemic stroke, large vessel occlusion, mechanical thrombectomy, perfusion, recanalization

## Abstract

Stroke is one of the leading causes of morbidity and mortality worldwide. Intravenous tissue plasminogen activator and mechanical thrombectomy comprise the two major treatments for acute ischemic stroke. Tissue plasminogen activator has been used for more than two decades and guidelines for hemodynamic management following tissue plasminogen activator administration are well established. However, mechanical thrombectomy is a relatively newer therapy and there is a paucity of evidence regarding hemodynamic management following large vessel occlusion strokes. The important tenets guiding the pathophysiology of large vessel occlusion strokes include understanding of cerebral autoregulation, collateral circulation, and blood pressure variability. In this narrative review, we discuss the current American Heart Association-American Stroke Association guidelines for the early management of acute ischemic stroke during different phases of the illness, encountered at different sections of a hospital including the emergency room, the neuro-interventional suite, and the intensive care unit. There is emerging evidence with regard to post-recanalization blood pressure management following large vessel occlusion strokes. Future research directions will include real-time blood pressure variability assessments, identifying the extent of impaired autoregulation, and providing guidelines related to range and personalized blood pressure trajectories for patients following large vessel occlusion strokes.

Stroke is the second leading cause of death affecting six million people every year worldwide (1). The global burden of ischemic stroke has increased by 37% between 1990 and 2010 (2). Per Centers for Disease Control and Prevention figures, stroke is the fifth leading cause of death and a leading cause of disability in the United States (US) (3). Strokes add 34 Billion USD to the US health care cost every year (4). Ischemic stroke is the most common subtype comprising 87% of all types of strokes (including subarachnoid and intracranial hemorrhages) in the US (4,5).

Intravenous tissue plasminogen activator (tPA) was US Food and Drug Administration (FDA) approved for the treatment of acute ischemic stroke in 1996 after the National Institute of Neurological Disorders and Stroke trial and has been the main stay of treatment ever since. After unsuccessful initial attempts involving intra-arterial treatments, the first endovascular clot retriever - Mechanical Embolus Removal in Cerebral Ischemia retriever was FDA-approved in 2004 (6,7). After a series of negative trials, largely attributed to study design errors, the Multicenter Randomized Clinical Trial of Endovascular Treatment for Acute Ischemic Stroke in the Netherlands showed the benefit of mechanical thrombectomy (MT) in large vessel occlusion (LVO) strokes in 2015 (7,8). This benefit was subsequently confirmed by several other LVO stroke trials including solitaire™ with the intention for thrombectomy as primary endovascular treatment, extending the time for thrombolysis in emergency neurological deficits - intra-arterial, endovascular treatment for small core and proximal occlusion ischemic stroke, endovascular revascularization with solitaire device versus best medical therapy in anterior circulation stroke within 8 hours, and trial and cost effectiveness evaluation of intra-arterial thrombectomy in acute ischemic stroke (8,9,10,11,12,13). MT has become the recommended standard for anterior circulation LVO strokes presenting within six hours of symptom onset, having a favorable radiological Alberta stroke program early computed tomography score and national institutes of health stroke scale 6 or greater (14). More recently, after the publication of the endovascular therapy following imaging evaluation for ischemic stroke 3 and clinical mismatch in the triage of wake up and late presenting strokes undergoing neurointervention with trevo (DAWN) trials in 2018, the time window for efficacy of MT was extended from16 to 24 hours, using perfusion imaging in a smaller group of patients (15,16).

MT for acute LVO strokes is a relatively new therapy compared to tPA and its peri-procedural guidelines have evolved with emerging evidence. We continue to learn about hemodynamic management following LVO strokes as we gather new data. In the interim, the American heart association/American stroke association (AHA-ASA) guidelines recommend blood pressure goals of lower than 180/105 mmHg following reperfusion, similar to the guidelines following tPA administration (14). However, the recanalization rates of these treatments are not similar. Studies have shown that tPA is able to achieve recanalization rates of 17%-38%, while MT is able to achieve 70%-90% recanalization (17,18,19). Therefore, similar BP goals cannot necessarily apply to both post-recanalization phase after tPA administration and MT. The purpose of this review was to examine the key concepts related to the patho-physiology of LVO strokes, current evidence on post-recanalization BP management, and to provide recommendations on the modified management strategies with regard to the current evidence.

## HEMODYNAMICS AND PATHOPHYSIOLOGY OF LVO STROKES

### Impaired autoregulation

**Figure 1:**
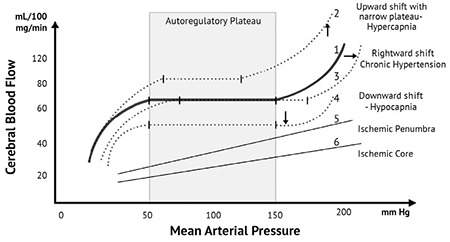
Cerebral autoregulation curve. Note the cerebral autoregulation curve (Curve 1) and autoregulatory plateau in the normal brain. The curve 2 depicts upward shift with a narrower plateau in hypercapnia, curve 3 depicts rightward shift in chronic hypertension, and curve 4 depicts a downward shift in hypocapnia. Plots 5 and 6 show the loss of autoregulation in ischemic penumbra and core, respectively [Meng and Gelb (23), and prepared by Dr. S. Das].

In a normal brain, blood vessels constrict in response to high pressures and dilate in response to low pressures, in order to maintain a constant perfusion to the cerebrovascular capillary bed (20,21). This phenomenon is called cerebral autoregulation and is an essential homeostatic process that allows the brain to receive adequate blood even in the setting of fluctuating systemic blood pressures, albeit, within a range of 60-150 mmHg (22). This range is shifted rightward in chronic hypertensive patients due to hypertrophy of the cerebral vessel walls (20). The autoregulatory curve shifts downwards in a setting of hypocapnia and shifts upwards with a narrow plateau in hypercapnia (23). Cerebral autoregulation is lost after an acute ischemic stroke (Figure 1) (24).

LVO strokes involve acute occlusion of blood vessels in the anterior circulation, such as the internal carotid, middle cerebral, and anterior cerebral arteries; as well as in the posterior circulation, such as the vertebral, basilar, and posterior cerebral arteries. The occlusion results from thrombosis in an atherosclerotic vessel or from an embolus originating from the heart or cervical blood vessels. After an acute stroke, cerebral autoregulation is known to be impaired, increasing the dependence of cerebral perfusion on the systemic pressures (25). Therefore, a peak in systemic blood pressure in the setting of pre-existing vaso-dilatation in the ischemic tissue places the infarcted tissue at risk of hemorrhagic transformation. This could result from luxury perfusion in the ischemic core, as well as from reperfusion injury following recanalization. Tissue with marginal blood flow called the penumbra that surrounds the ischemic core is also susceptible to these changes (26,27,28). On the other hand, brief and drastic blood pressure drops in the first 24 hours of an acute ischemic stroke has been shown to contribute to the loss of the penumbral tissue (29). Interestingly, the blood vessels on the contralateral side of the ischemia are also dilated in order to perfuse the penumbra through the collateral circulation. Cerebrovascular pressure reactivity and tissue oxygenation measurements using near-infrared spectroscopy have been reported to estimate autoregulation index in stroke patients (30,31,32).

### Collateral circulation

During ischemic stroke, although the core of the infarct undergoes irreversible damage, the penumbra in the periphery of the infarct can be salvaged (22). The penumbra receives blood flow via collaterals. The collateral circulation has an anatomic aspect, as well as a physiologic aspect. The anatomic aspect of the collateral vessels can be quantified using a multiphase CT angiogram or a conventional cerebral angiogram. A multiphase CT angiography collateral score ranges from 0 indicating no visible collateral vessels to a maximum of 3 indicating extensive collateral vessels (33,34). The collateral circulation is dynamic and its physiological aspect is difficult to quantify. A radiographic filling delay of one second or less during contrast injection in a diagnostic angiogram indicates the presence of a robust collateral circulation (33,35,36). The extent of the collateral circulation depends on genetic factors, the chronicity of the vascular lesion, physiologic parameters such as perfusion pressure, and the capacity for autoregulatory dilatation. For example, an elderly patient with long standing atherosclerosis may have extensive anatomic collaterals depending on the chronicity of the lesion. However, in contrast, a younger patient may have a better physiological collateral circulation due to a greater capacity for autoregulatory dilatation. A perfusion scan reveals the adequacy of collaterals to keep the ischemic tissue viable. The presence of extensive and robust collaterals have been associated with a higher likelihood of recanalization and reduced infarct volume, even with treatment failure (37,38,39,40).

### Blood pressure variability

Blood pressure variability in the setting of an acute ischemic stroke is due to a complex interplay between autonomic regulation and arterial wall mechanical properties (41). Over 60% of patients with acute ischemic strokes experience post-infarct hypertension; blood pressure peaks and fluctuations are clinically difficult to interpret due to an existing evidence gap regarding the expected blood pressure trajectory for each degree of recanalization (42,43,44,45,46). For example, incomplete recanalization may result in increased intracranial vascular resistance and subsequent blood pressure variability (47). Likewise, patients who present with less blood pressure variability experience better short-term (48) and long-term (49) functional outcomes. Blood pressure variability can be measured using several statistical formulas, including standard deviation (SD), coefficient of variation, successive variation (SV), and average real variation (ARV). Unfortunately, even time-weighted averages of relatively high-frequency BP data appear to be insufficient in presenting the successive variability. While a few measures like SD are dependent on mean blood pressure, parameters like SV and ARV take into account the point to point variations (50). These parameters also take into account the rapid peaks in BP (51,52).

### Blood pressure trajectory

A study evaluating the natural history of blood pressure following MT in 68 patients with LVO strokes was reported in 2016 (53). It was observed that there is an initial drop in BP in all patients within the first 8-12 hours, followed by a plateau phase of a steady BP trajectory. The initial drop was greater in the recanalized patients compared to the non-recanalized patients (53). Recent reports have identified five distinct blood pressure trajectories following LVO strokes and have proposed how these trajectories determine the 90-day functional outcomes in patients. These trajectories were named as low, moderate, moderate-to-high, high-to-moderate, and high. The last two trajectories were associated with higher odds of an unfavorable 90-day outcome (52). Higher blood pressure trajectories may represent either a re-occlusion or an impaired perfusion dynamic in the affected tissue.

## CURRENT GUIDELINES

**Figure 2:**
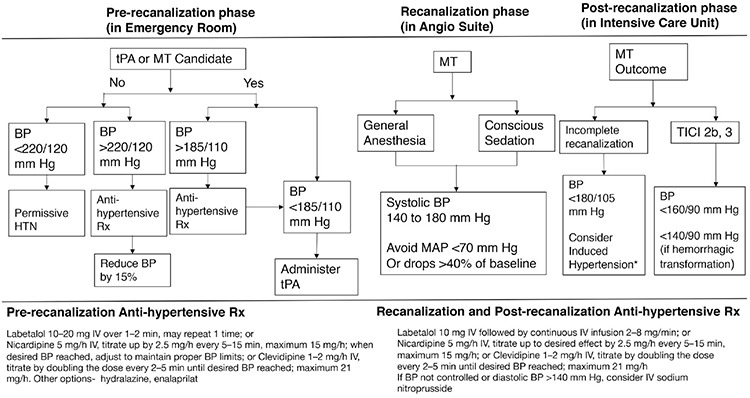
Blood Pressure goals and anti-hypertensive treatment options in different phases of a Large Vessel Occlusion stroke. (tPA- tissue Plasminogen Activator, BP- Blood Pressure, MT- Mechanical Thrombectomy, TICI- Thrombolysis in Cerebral Infarction Score, Rx- Treatment) [Vitt et al. (68), and prepared by Dr. S. Das].

Due to the distinct pathophysiology underlying the problem, it will be most appropriate to discuss the current guidelines in terms of the three phases following an acute ischemic stroke: (a) pre-recanalization phase or initial assessment phase encountered in the emergency department, (b) recanalization phase or procedure phase, when the patient is in the angiography suite for an interventional neuro-vascular approach to treat the thrombus, and (c) early post-recanalization phase, the first 24 hours following thrombectomy, when the patient is managed in the ICU after the procedure (Figure 2).

### Pre-recanalization phase (initial assessment/admission phase)

Hypotension and hypovolemia should be prevented to maintain systemic perfusion and support end-organ function (54). A drop in systolic blood pressure (SBP) of >50 mmHg over a 24-hour period or an acute drop of >30 mmHg may worsen the overall outcomes (55). In this study, acute drop in BP was defined as the largest drop in BP between a measurement and one immediately preceding it. BP data was acquired every 15 minutes in the first 2 hours after presentation, every 30 minutes in the next 6 hours, and hourly until the first 24 hours after presentation (55). Also, in another study, a drop in systolic blood pressure to less than 110 mmHg was shown to result in increased mortality (56).

The 2019 AHA/ASA guidelines for the early management of acute ischemic stroke introduced a few new recommendations regarding blood pressure management. The recommendations discussed in this section are class I recommendations unless stated otherwise. Table 1 summarizes the meaning of each level of recommendation. In patients presenting with higher blood pressures, the blood pressure should be lowered to <185/110 mmHg before fibrinolytic treatment. A class IIa recommendation proposed that blood pressure should be maintained at ≤180/105 mmHg for patients with planned MT, who did not receive tPA. The efficacy of drug-induced hypertension is not established (class IIb). After tPA treatment, the blood pressure should be maintained at <180/105 mmHg for the first 24 hours (54). In patients with presenting blood pressure ≥220/110 mmHg, it is customary to identify any co-morbid conditions including concomitant acute coronary event, acute heart failure, aortic dissection, postfibrinolysis symptomatic intracranial hemorrhage, or preeclampsia/eclampsia, which will require lowering blood pressure. In the absence of any of the above comorbidities, the benefit of initiating or reinitiating anti-hypertensive treatment is not reported. Therefore, AHA/ASA guidelines propose a class IIb recommendation to lower the blood pressure only minimally, <15% of baseline in these patients. For patients who present with blood pressure <220/110 mmHg, who did not receive tPA or MT and did not have any comorbidities, the guidelines recommend against lowering the blood pressure from baseline (Figure 2) (54).

### Recanalization phase (mechanical thrombectomy procedure phase)

The Society of Neuroscience in Anesthesiology and Critical Care recommends that the SBP be maintained between 140 and 180 mmHg during MT (57). Abrupt drops in blood pressure > 40% of the baseline as well as a drop in the mean arterial pressure (MAP) below 70 mmHg have been associated with poor outcomes (58). The type of anesthesia used during MT could possibly confound these outcomes. Initial retrospective observational studies suggested that conscious sedation may yield better outcomes when compared to general anesthesia, which may delay recanalization (59,60,61). However, in recent prospective trials, where general anesthesia and conscious sedation were compared in randomized studies such as sedation vs intubation for endovascular stroke treatment and general or local anesthesia in intra-arterial therapy, general anesthesia was associated with a higher rate of favorable 90-day functional outcomes. Even though the time to groin puncture was slightly higher with general anesthesia in the GOLIATH trial, the overall time to recanalization was improved (62,63,64). Looking pragmatically, as long as there is an aggressive blood pressure control as described above, general anesthesia and conscious sedation may remain in clinical equipoise for procedural anesthesia care during MT.

### Post-recanalization phase

In the absence of a randomized controlled trial data for post-recanalization BP management following MT, AHA-ASA guidelines propose a class II recommendation to keep SBP ≤180/105 mmHg during the procedure and in the first 24 hours following the procedure. For completely recanalized patients, the SBP could be kept <180/105 mmHg (class II b) (54). In spite of the guidelines, the current practice is to keep the SBP ≤140 mmHg to 160 mmHg as used in the DAWN trial (15,65,66). Also, a more intensive BP control is intuitively pursued in patients with adjunct extra-cranial or intracranial lesions treated with stents to minimize reperfusion injury (67).

AHA/ASA guidelines-based SBP goal is arbitrarily set higher in patients with incomplete reperfusion to ensure perfusion to the penumbra through collaterals. However, we now see emerging evidence for post-recanalization BP management that may require incorporation into the subsequent AHA/ASA guidelines.

An initial retrospective study in 2017 reported that higher blood pressures may relate to worse outcomes and a 10 mmHg increase in maximum SBP within the first 24 hours after MT was an independent predictor of both worse functional independence and increased mortality at 3 months (66). This study also found that moderate blood pressure control <160/90 mmHg during the first 24 hours post-MT was associated with the lowest 3-month mortality, when compared to the groups with intensive blood pressure control <140/90 mmHg as well as those with permissive hypertension (BP <180/105 mmHg following tPA or MT, or <220/110 mmHg in patients without any acute intervention) (66). The limitation of this study was that it was a retrospective study and it was unclear if blood pressure was the cause of the poor outcomes or a mere indicator. The first multicenter prospective study in this regard is the Blood Pressure after Endovascular Therapy for Ischemic Stroke, BEST trial which enrolled 485 patients in 12 centers across the US from November 2017 to July 2018. The study showed that a peak SBP of >158 mmHg within the first 24 hours post recanalization increased the likelihood of a poor 90-day functional status (51). Most patients in this sample were fully recanalized [thrombolysis in cerebral infarction score (TICI) 2b or 3 score]. There is no clear evidence regarding blood pressure goals in partially recanalized or non-recanalized patients (68). Intuitively, these patients may require higher blood pressure goals to ensure perfusion to the penumbra. Our group has demonstrated poor discharge outcomes among successfully recanalized patients who had sustained hypoperfusion for >12 hours, as well as those with higher blood pressure variability in the first 24 hours following MT (69,48). Similarly, in a post-hoc analysis of the BEST trial data, the patients with higher blood pressure variability within the first 24 hours had poor 90-day functional outcomes (49).

### Blood pressure measurement and anti-hypertensives

As per AHA/ASA guidelines, blood pressure should be measured every 15 minutes in the first 2 hours following tPA or MT, every 30 minutes until the first 6 hours, and only hourly thereafter (14). AHA/ASA recommends a few fast-acting anti-hypertensives with a short-lasting effect to reach the above discussed blood pressure goals. These include intravenous scheduled bolus doses of Labetalol, Hydralazine, or Enalaprilat and continuous drip forms of Nicardipine and Clevidipine (54). These anti-hypertensives have demonstrated clinical equipoise and therefore, selection may be based on their availability. Simultaneously, escalating oral or per tube use of anti-hypertensives allows blood pressure to be maintained within the goal range recommended. For blood pressure levels refractory to these treatments or diastolic blood pressure >140 mmHg, intravenous Nitroprusside can be used in the continuous drip form (54).

On the other hand, we acknowledge that lower blood pressure levels can also be harmful. There are ongoing trials to examine the effect of induced hypertension using peripherally acting vasopressors like phenylephrine to ensure better perfusion to the penumbra. There is no conclusive data in favor of such treatment (class IIb), meaning it is possible to improve low blood pressure levels with vasopressors; however, the effects of pressor-based normalized blood pressure on clinical outcomes are not known at this point (54).

## SUMMARY of RECOMMENDATIONS

The current AHA/ASA guidelines recommend a blood pressure <185/110 mmHg before fibrinolytic treatment and that blood pressure be maintained at ≤180/105 mmHg in the first 24 hours thereafter, if the patient received tPA and/or MT. However, in the light of BEST trial and reflecting on the parameters used in the DAWN trial, we propose the following Blood Pressure goals.

(a) For patients who are not recanalized (TICI 0), blood pressure limits of ≤180/105 mmHg should be permitted if these patients received tPA. One can be more liberal regarding blood pressure if these patients did not receive tPA and attempt to reduce the blood pressure by 15% of the baseline only if the presenting blood pressure is greater than 220/110 mmHg. In patients who did not receive tPA and could not be recanalized, induced hypertension is a consideration.

(b) For patients who are partially recanalized (TICI 1, 2a), the blood pressure goal should be ≤160/90 mmHg. Higher SBP up to 180 mmHg may be permitted in cases of neurological deterioration. It is noteworthy that higher blood pressure variability and disruption of autoregulation may persist longer in these patients (67,68). Acute blood pressure drops in this group of patients may be detrimental.

(c) For patients who are completely recanalized (TICI 2b, 3), blood pressure goals should be ≤160/90 mmHg. Greater blood pressure variability may be a predictor of poor outcomes in these patients. Therefore, acute peak and fall in blood pressure should be avoided in this group.

(d) More intensive blood pressure goals (<140 mmHg) may be required for patients who are fully recanalized but have symptomatic intracerebral hemorrhage (ICH) or ICH on imaging.

A significant but slow drop in blood pressure is seen in the early post-recanalization phase and more in successfully recanalized patients as compared to non-recanalized patients. Thereafter, a plateau in blood pressure is achieved after 10-12 hours of recanalization. The physiological mechanism behind this phenomenon is unclear (70,71). There is emerging evidence that blood pressure trajectories following recanalization may be guided by the level at which autoregulation is functioning. While traversing these trajectories, the blood pressure varies from point to point. Post-recanalization phase blood pressure variability has been shown to determine the discharge disposition, as well as the 90-day functional outcomes.

Hemodynamic management of LVO stroke patients require close blood pressure monitoring and its careful management for at least the first 24 hours after MT. Emerging research utilizing continuous and noninvasive methods to identify cerebral autoregulatory ranges and the use of multimodal monitoring to assess real-time blood pressure variability, may guide the personalized management of blood pressure. In this narrative review, we aimed to summarize the blood pressure management guidelines for the care of LVO stroke patients with specific emphasis on blood pressure variability.

## Figures and Tables

**Table 1:**
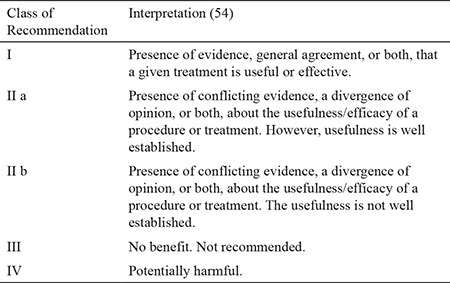
Classes of recommendation

**Table 2:**
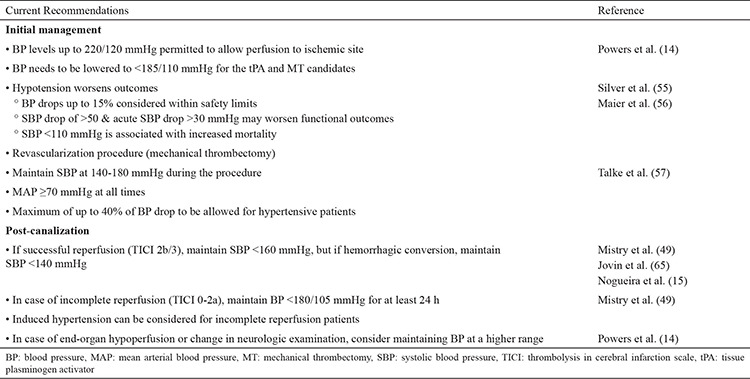
Blood pressure management in large vessel occlusion strokes
